# The RIG-I ATPase core has evolved a functional requirement for allosteric stabilization by the Pincer domain

**DOI:** 10.1093/nar/gku817

**Published:** 2014-09-12

**Authors:** David C. Rawling, Andrew S. Kohlway, Dahai Luo, Steve C. Ding, Anna Marie Pyle

**Affiliations:** 1 Department of Molecular Biophysics and Biochemistry, Yale University, New Haven, CT 06520, USA; 2 Department of Molecular, Cellular, and Developmental Biology, Yale University, New Haven, CT 06520, USA; 3 Howard Hughes Medical Institute, Chevy Chase, MD 20815, USA; 4 Department of Chemistry, Yale University, New Haven, CT 06520, USA

## Abstract

Retinoic acid-inducible gene I (RIG-I) is a pattern recognition receptor expressed in metazoan cells that is responsible for eliciting the production of type I interferons and pro-inflammatory cytokines upon detection of intracellular, non-self RNA. Structural studies of RIG-I have identified a novel Pincer domain composed of two alpha helices that physically tethers the C-terminal domain to the SF2 helicase core. We find that the Pincer plays an important role in mediating the enzymatic and signaling activities of RIG-I. We identify a series of mutations that additively decouple the Pincer motif from the ATPase core and show that this decoupling results in impaired signaling. Through enzymological and biophysical analysis, we further show that the Pincer domain controls coupled enzymatic activity of the protein through allosteric control of the ATPase core. Further, we show that select regions of the HEL1 domain have evolved to potentiate interactions with the Pincer domain, resulting in an adapted ATPase cleft that is now responsive to adjacent domains that selectively bind viral RNA.

## INTRODUCTION

Cellular pattern recognition receptors (PRRs) protect against host invasion by recognizing specific pathogen associated molecular patterns and initiating a defensive immune response (1–7). Retinoic acid-inducible gene I (RIG-I) belongs to a class of these intracellular RNA sensors, which include melanoma differentiation-associated gene 5 (MDA5) and laboratory of genetics and physiology 2 (Lgp2), together comprising the RIG-I like receptor (RLR) family of PRRs (8–11). The RIG-I protein is built around a central DExD/H-box core comprised of two RecA-like folds, HEL1 and HEL2, with the latter containing a specialized insertion domain (HEL2i) that facilitates recognition of dsRNA (12–17). In addition to the ATPase core, RIG-I also contains a C-terminal domain (CTD) that enhances specificity for pathogenic RNA by recognizing the terminal 5′ triphosphate moiety (18–20) on genomic RNA (21,22) or replicative intermediates (23) of numerous RNA viruses such as influenza, flaviviruses, hepatitis C virus and Sendai virus. Upon dsRNA binding, immune signaling is initiated through interactions between the caspase activation and recruitment domains (CARDs) at the N-terminus of RIG-I and an adaptor protein known as MAVS (8,24–27).

RIG-I surveys the cytoplasm in a silent state, whereby the CARDs are sequestered through intramolecular interactions with the HEL2i domain (8,13). Upon infection, RIG-I binds viral RNA through a series of multidomain interactions involving the CTD, the ATPase core and the HEL2i domain. Most significantly, the CTD caps the dsRNA terminus, binding with enhanced affinity in the presence of a 5′triphosphate moiety (18,28), while HEL1, HEL2 and HEL2i wrap around the duplex stem to form a nearly complete ring around the RNA (12–14,29). The interactions with RNA serve to orient the HEL1 and HEL2 domains and promote formation of an active site pocket that catalyzes ATP binding and hydrolysis (13,28,30,31). During this process, the CARDs are expelled from HEL2i into solution, making them available for covalent ubiquitination by TRIM25 (32) or non-covalent binding with free K63-linked tetra-ubiquitin chains (33,34), and subsequent activation of MAVS. Despite extensive structural and biochemical analysis, the precise molecular mechanism by which downstream signaling is accomplished remains unclear. Unanswered questions relevant to this process involve the oligomerization state of RIG-I during signaling, and whether translocation is biologically relevant to RIG-I function (35–43). Nevertheless, it is clear that RIG-I prefers shorter RNA duplexes than its counterpart MDA5 (44–46), and that interaction with these ligands facilitates intramolecular rearrangements that aid in downstream signaling.

**Figure 1.**
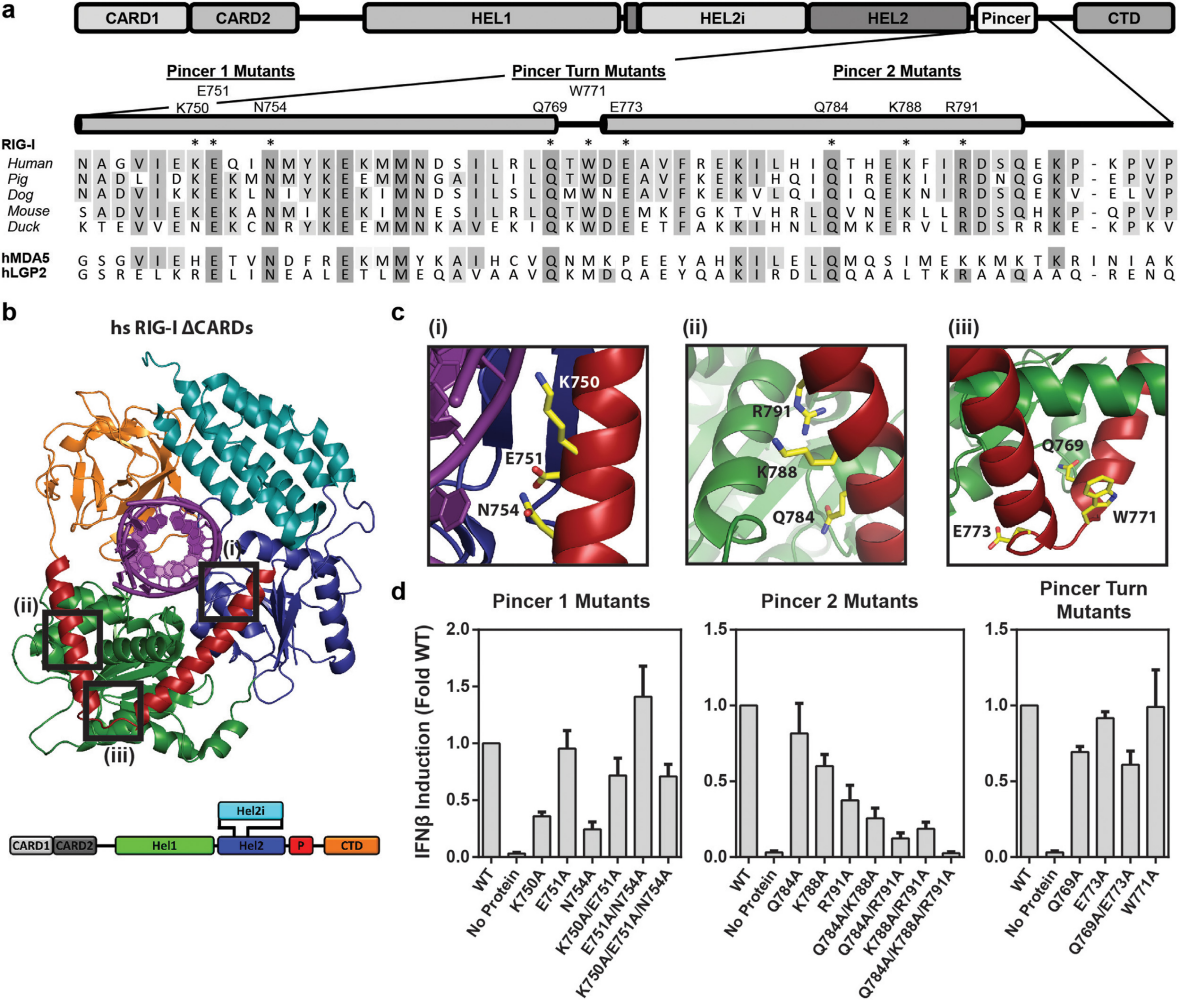
Sequence and structural organization of the RIG-I Pincer domain. (**a**) Amino acid sequence alignment for the RIG-I Pincer domain, including selected protein orthologs and paralogs; shading reflects sequence identity, with darker tint denoting a higher degree of conservation. Residue groups selected for mutation are listed above the alpha helices (shown as gray bars) and denoted by asterisks above the sequence. (**b**) Three-dimensional structure of human RIG-I bound to a 14 bp RNA duplex (PDB: 3TMI). Boxes indicate sites of pincer mutations and are labeled to correlate with the panels in (**c**). Close up views of Pincer residues selected for mutation: left panel, mutations in the N-terminal Pincer helix (Pincer 1 mutants); center panel, mutations in the C-terminal Pincer helix (Pincer 2 mutants); right panel, mutations at the turn between the two helices (Pincer turn mutants). (**d**) Immune signaling by Pincer mutant proteins. Each plot represents the mean IFN-β signaling activity of Pincer mutant constructs normalized to wild type signaling behavior, with error bars representing the standard deviation across three experiments. Constructs are grouped by their location within the Pincer domain: left panel, Pincer 1 mutants; center panel, Pincer 2 mutants; right panel, Pincer turn mutants.

In addition to the CARDs, the ATPase core and CTD, structural studies of RIG-I have revealed a novel alpha-helical domain termed the Pincer or Bridging domain, which is pronounced and uniquely conserved among RLR members of helicase superfamily 2 (SF2) (11–14,28). The Pincer consists of two alpha-helical segments that follow HEL2, stacking back across HEL1 and leading into the CTD via a proline-rich linker (Figure 1a and b). The conservation and orientation of the Pincer suggests that it plays an important role in RLR function; this is underscored by the observation that the RecA-like fold of HEL1 contains unusual adaptations, such as changes in the size and orientation of secondary structural elements—including the α-helical axel motif—which facilitate multiple non-covalent interactions with the Pincer helices (47). However, despite its pronounced influence on RIG-I architecture, little is known about the functional significance of the Pincer domain with regard to ligand binding, allosteric control, catalysis and signaling activities of RIG-I (48).

To delineate the importance of the Pincer domain in RIG-I function, we perturbed the molecular interfaces between the Pincer domain and other regions of the molecule (Figure 1a and b). We then quantitatively assessed the impact of these perturbations on the RNA binding, ATPase and signaling activities of RIG-I. Given the apparent adaptations in HEL1 that mediate interactions with the Pincer, we focused on understanding how decoupling of these domains might influence the functional activities of the enzyme. We show that disruption of the Hel1-Pincer interface impairs the ability of RIG-I to engage in immune signaling, and we show that this arises from defects in both RNA binding and ATP hydrolysis. Further, we show that these defects are due to allosteric destabilization of the ATPase active site, indicating that a functional Pincer is required for structural support and activation of the ATPase core of RLR proteins. This mechanical design ensures that information on viral RNA binding is specifically relayed to the enzymatic and signaling domains of the protein, resulting in sensitive detection of viral RNA and discrimination among RNA molecules in the cell.

## MATERIALS AND METHODS

### Cloning, expression and purification of recombinant RIG-I

Wild-type *hs* RIG-I was cloned into the pET-SUMO vector (Life Technologies) using the manufacturer's protocols. All mutations were introduced into the parent plasmid using the QuikChange II kit (Agilent Technologies). For expression, plasmids were transformed into Rosetta II(DE3) *Escherichia coli* cells (Novagen) and grown in LB media supplemented with 50 mM Potassium Phosphate pH 7.4 and 1% glycerol. Expression was induced by the addition of isopropyl-β-D-thiogalactopyranoside (IPTG) to a final concentration of 0.5 mM. Cells were grown for 24 h at 16°C, then harvested by centrifugation, resuspended in lysis buffer (20 mM Phosphate pH 7.4, 500 mM NaCl, 10% glycerol, 5 mM β-mercaptoethanol (βME)) to a final volume of 50 ml and frozen at −80°C. For lysis, frozen pellets were thawed at room temperature, then resuspended in an additional 200 ml lysis buffer per 4L pellet. Cells were lysed by passage through a microfluidizer at 15,000 psi, and the lysate was clarified by ultracentrifugation at 100,000×g for 30 min. Soluble lysate was incubated on 2.5 ml Ni-NTA beads (Qiagen), washed with lysis buffer containing an additional 40 mM imidazole, then eluted in Ni elution buffer (25 mM HEPES pH 8.0, 150 mM NaCl, 220 mM Imidazole, 10% glycerol, 5 mM βME). Eluted protein was bound to a HiTrap Heparin HP column (GE Biosciences), washed in buffer containing 150 mM NaCl and eluted stepwise at 0.65 M NaCl. The SUMO tag was then removed by incubation with SUMO protease for 2 h at 4°C. Finally, monomeric protein was collected by passage over a HiPrep 16/60 Superdex 200 column (GE Biosciences) in gel filtration buffer (25 mM MOPS pH 7.4, 300 mM NaCl, 5% glycerol, 5 mM βME). Peak fractions were concentrated to 10–20 μM using a centrifugal concentrator with a 50 kD molecular weight cutoff (Millipore). Concentrations were determined spectrophotometrically using an extinction coefficient of ϵ = 99,700 M^−1^ cm^−1^ at λ = 280 nm. Protein preparations were aliquoted, flash frozen using liquid nitrogen and stored at −80°C.

### HEK 293T cell culture and IFN-β induction assays

The pUNO-hRIG-I vector containing wild type RIG-I for expression in mammalian cell culture was purchased from Invivogen. All mutations were introduced into the parent plasmid using the QuikChange II kit (Agilent Technologies).

HEK 293T cells—chosen because they do not express RIG-I endogenously (proteinatlas.org)—were grown and maintained in 10 cm dishes containing Dulbecco's Modified Eagle Medium (Life Technologies) supplemented with 10% heat-inactivated fetal calf serum (Hyclone) and Non-Essential Amino Acids (Life Technologies). For IFN-β induction assays, 0.5 ml of cells at 100,000 cells/ml was seeded in 24-well plates. After 24 h, each well of cells was transfected with 3 ng WT or mutant pUNO-hRIG-I, 6 ng pRL-TK constitutive Renilla luciferase reporter plasmid (Promega) and 150 ng of an IFN-β/Firefly luciferase reporter plasmid using the Lipofectamine 2000 transfection reagent (Life Technologies) per the manufacturer's protocol. Protein expression was allowed to proceed for 24 h, at which point the cells were challenged by transfection of 1 μg of the synthetic dsRNA analog poly I:C (Invivogen), also using the Lipofectamine 2000 reagent. After 12–16 h, cells were harvested for luminescence analysis.

To assess IFN-β induction using a dual luciferase assay, cells were harvested and lysed as follows: Growth media was aspirated from each well, and 100 μl of passive lysis buffer (Promega) was added. Lysis proceeded for 15 min at room temperature. The lysates were clarified by centrifugation, and 20 μl samples of the supernatant were transferred to a 96-well assay plate for analysis using the Dual-Luciferase Reporter Assay System (Promega). Luminescence was measured using a Biotek Synergy H1 plate reader. The resulting Firefly luciferase activity (i.e. the induction of IFN-β) was normalized to the activity of the constitutively expressed Renilla luciferase to account for differences in confluency and transfection efficiency across sample wells.

### Fluorescent RNA binding assay

**Figure 2.**
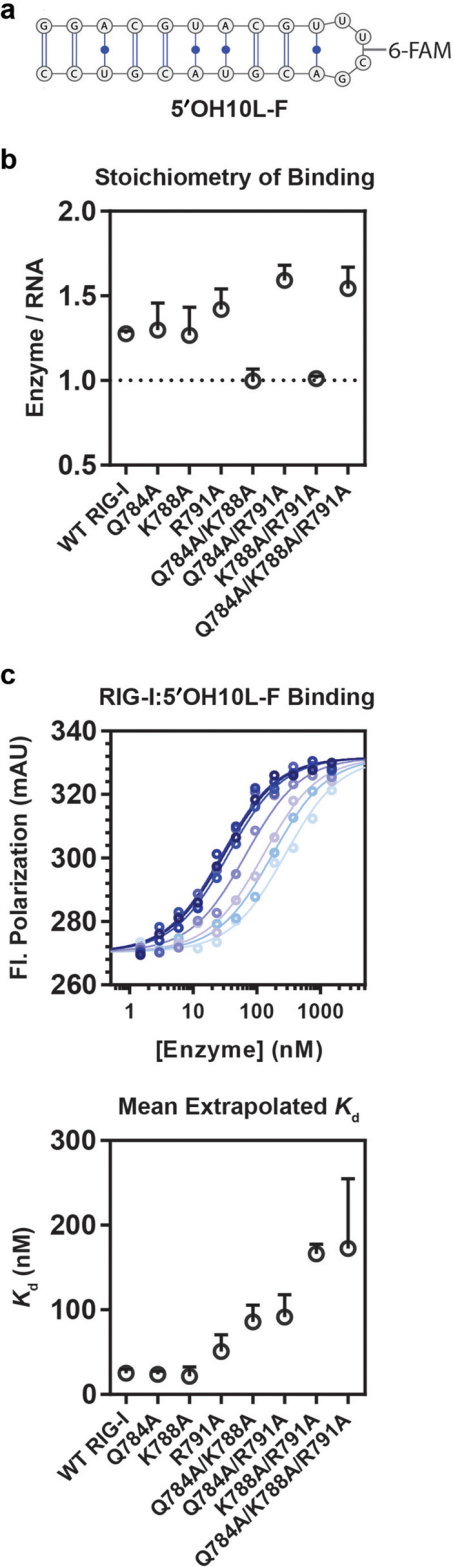
RNA binding is impaired in Pincer 2:HEL1 mutant proteins. (**a**) Schematic diagram representing the sequence and structure of the 5′OH10L-6-Carboxyfluorescein ligand (5′OH10L-F). The fluorescent modification is chemically conjugated to the substrate through the phosphate backbone between the central bases of the tetraloop. (**b**) Binding stoichiometry of RIG-I and 5′OH10L-F, studied by fluorescence polarization. The plot describes the mean ratio of [enzyme] to [RNA] for each construct, with error bars denoting the standard deviation across three experiments. (**c**) RNA binding by RIG-I was measured using a fluorescence polarization assay in which 1 nM 5′OH10L-F was incubated in the presence of wild type or mutant RIG-I at concentrations varying from 0 to 1.5 μM. Upper panel: Polarization values were plotted as a function of enzyme concentration and fit to Equation (2). The color gradient denotes accumulation of mutations, with double and triple mutants shown in lighter shades. Lower panel: Mean equilibrium dissociation constants extrapolated from three experiments are plotted with error bars indicating the standard deviation across independent fits.

The fluorescent RNA hairpin used in binding experiments (TriLink Biotech) contained a 10 base pair duplex capped by a tetraloop with the sequence GGACGUACGUUU(6-FAM)CGACGUACGUCC and included an internal fluorescent modification, carboxyfluorescein (Figure 2a). Binding assays were carried out in 384-well plate format. Briefly, dsRNA was diluted into binding buffer (25 mM MOPS pH 7.4, 150 mM KCl, 2 mM DTT, 2 mM MgCl, 0.01% Triton X-100) to a concentration of 2 nM. The RIG-I protein of interest was then diluted into binding buffer over a 12-pt series of concentrations and mixed 1:1 with RNA samples (final RNA concentration of 1 nM) to a volume of 20 μl. Final RIG-I concentrations varied from 1.5 to 1500 nM. Samples were equilibrated at room temperature for 1 h. Fluorescence polarization was measured using a Biotek Synergy H1 plate reader. Samples were excited through a bandpass filter at 485/20 nm and fluorescence emission was measured through a bandpass filter at 528/20 nm. Polarization was calculated using the following equation:

(1) \begin{equation*} P = \frac{{I_{||} - G*I_ \bot }}{{I_{||} + G*I_ \bot }} \end{equation*}

Where *I*_∥_ is the intensity of the fluorescent light parallel to the plane of excitation, *I*_⊥_ is the intensity of fluorescent light perpendicular to the plane of excitation and *G* is an empirically determined correction factor accounting for instrumental bias toward the detection of horizontally polarized light; in this case *G* = 0.87.

An individual experiment consisted of two replicates of each protein concentration for which polarization measurements were taken two or three times, yielding four to six values for each condition. The mean polarization values were then plotted against protein concentration and fit to a one-site total binding Equation (2):

(2) \begin{equation*} y = y_0 + \frac{{y_{\max } *x}}{{K_d + x}} \end{equation*}

Where *y*_0_ represents the polarization value when [enzyme] = 0 nM, *y*_max_ represents the polarization achieved at a saturating enzyme concentration and *K*_d_ is the dissociation constant. Three experiments were performed for each RIG-I construct, with each reported *K*_d_ value representing the mean and standard deviation across these experiments.

### NADH coupled ATPase activity assay

RIG-I ATPase activity was measured using an established absorbance-based coupled assay system. The RIG-I protein of interest was diluted into ATPase assay buffer (25 mM MOPS pH 7.4, 150 mM KCl, 2 mM DTT, 0.01% Triton X-100) to a final concentration of 5 nM for *K*_M, RNA_ experiments or 10 nM for *K*_M, ATP_ experiments in the presence of a coupled assay mix (1 mM NADH, 100 U/ml lactic dehydrogenase, 500 U/ml pyruvate kinase, 2.5 mM phosphoenol pyruvic acid).

For *K*_M, ATP_ experiments, protein samples were incubated for at least 2 h at room temperature in the presence of 1 μM RNA. A 1:1 ATP/MgCl_2_ mix was then diluted into assay buffer over a 12-pt, 3:2 dilution series resulting in final concentrations varying from 30 to 5000 μM. Reactions were initiated by adding the ATP/MgCl_2_ mix to the protein/NADH/RNA sample mix.

For *K*_M, RNA_ experiments, the RNA of interest was diluted into assay buffer over a 12-pt concentration series and added to the protein/NADH sample mix resulting in RNA concentrations varying from 1 to ∼1000 nM. Samples were incubated for at least 2 h at room temperature. The reaction was initiated by the addition of 5 mM ATP/5 mM MgCl_2_ to all wells.

The rate of ATP hydrolysis was determined indirectly by monitoring the conversion of NADH to NAD^+^ that results in a loss of sample absorbance at 340 nM. The assay was performed in 96-well format and absorbance was measured over a 10 min time course using a Biotek Synergy H1 Plate Reader. Mean velocities were extrapolated for each time course and plotted as a function of either ATP or RNA concentration. These data were then fit to the quadratic solution of the Briggs–Haldane equation:

(3) \begin{equation*} \begin{array}{*{20}l} {y = y_0 + ({\rm amp}) *} {\frac{{x + p + K_M - \sqrt {(x + p + K_M )^2 - 4xp} }}{{2p}}} \\ \end{array} \end{equation*}

Where *y*_0_ = basal activity, defined as background catalytic velocity observed in the absence of RIG-I, amp = *v*_max_ – *y*_0_ = *k*_cat_, *x* = total ATP or RNA concentration, *p* = total protein concentration and *K*_M_ is the Michaelis constant for the variable substrate.

### *In silico* structural analyses

Protein tertiary structure comparisons were performed using the Dali server at http://ekhidna.biocenter.helsinki.fi/dali_server/start. PDB 4A36 was used as the query structure. Results were sorted by Z-score, a statistical method describing goodness of fit defined in Ranjith-Kumar *et al.* (48). From these results, 22 structures representing unique proteins were selected based on a cutoff of *Z* ≥ 15. These structures were then used in primary and secondary structural alignments which were also performed using the Dali server. No structures fitting the above criteria were excluded. The selected window depicted in Figure 4a contains a portion of the full secondary structure alignment encompassing residues 383–433 of *hs* RIG-I.

**Figure 3.**
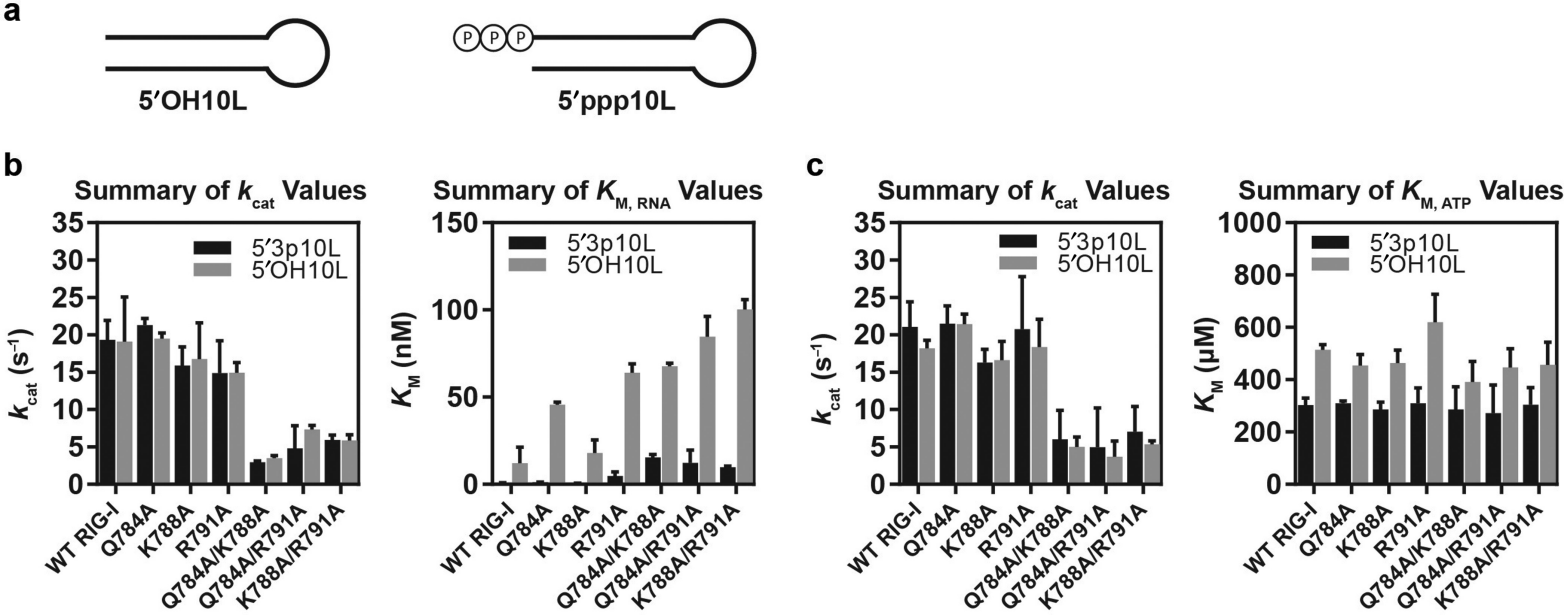
Pincer 2:HEL1 mutants exhibit diminished ATPase rates and increased *K*_M, RNA_. (**a**) Schematic of the 10L RNA hairpins used in the study, containing either a 5′ triphosphate or 5′ hydroxyl terminus. (**b**) Summary of steady-state kinetic parameters for Pincer:HEL1 mutants (obtained as a function of varying RNA concentrations). Left panel: summary of *k*_cat_ values obtained for all RIG-I constructs stimulated by both RNA ligands. Right panel: summary of *K*_M, RNA_ values obtained for all RIG-I constructs stimulated by both RNA ligands. (**c**) Summary of steady-state kinetic parameters for Pincer:HEL1 mutants (obtained as a function of varying ATP concentrations). Left panel: summary of *k*_cat_ values obtained for all RIG-I constructs stimulated by both RNA ligands. Right panel: summary of *K*_M, ATP_ values obtained for all RIG-I constructs stimulated by both RNA ligands. All values represent the mean of three experiments, with error bars representing the standard deviation across these values.

**Figure 4.**
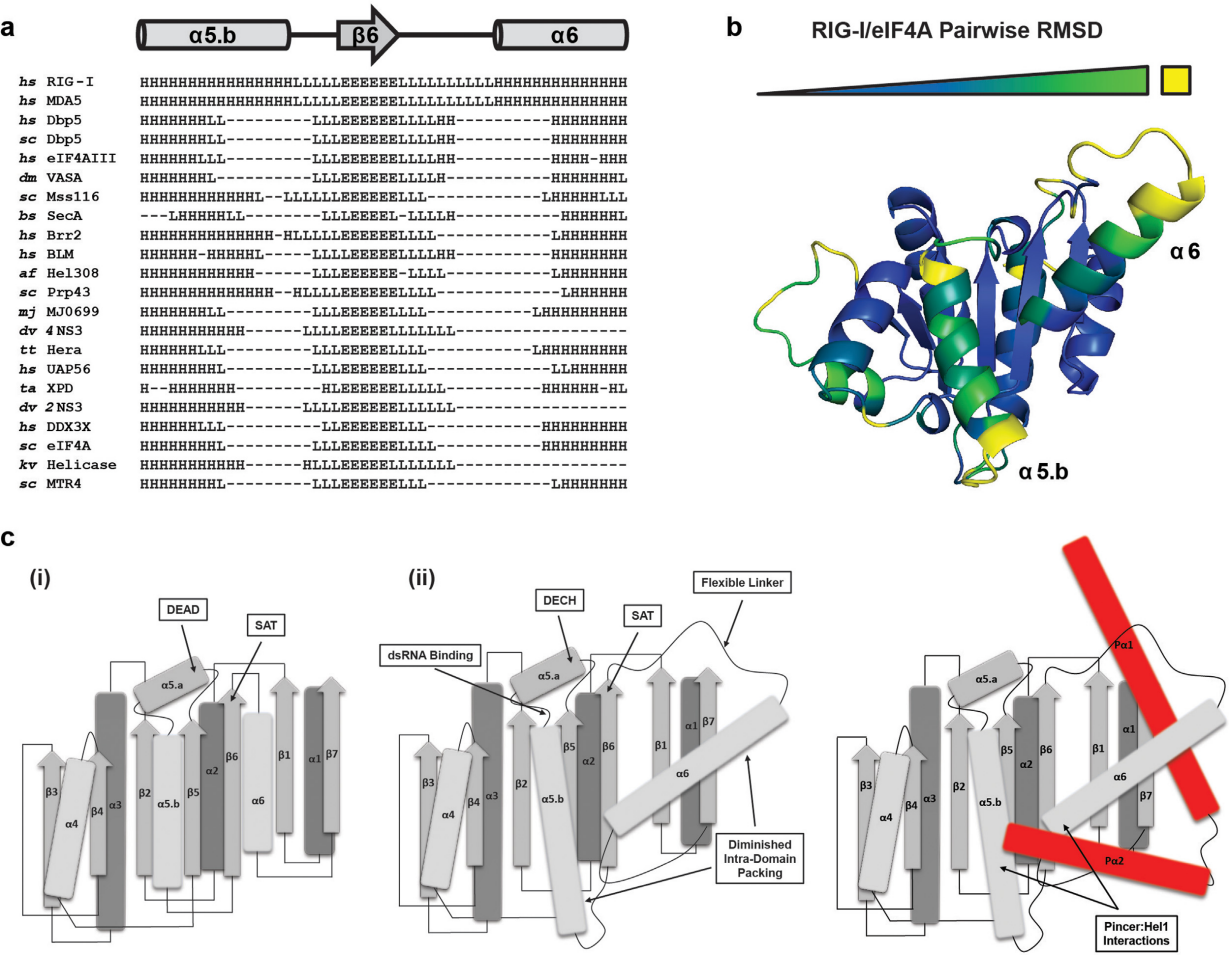
Unusual structural features observed within the HEL1 domain of RIG-I. (**a**) Secondary structural alignment between RIG-I and related proteins as identified by the Dali server. The alignment window encompasses residues 383–433 of RIG-I. (**b**) The RecA domain containing conserved motifs Q, IA, IB, II and III from eIF4a was globally aligned with the HEL1 domain of RIG-I. The backbone RMSD of HEL1 from the eIF4a RecA domain was calculated for the alignment and is represented on the structure by a color gradient from blue to green, with green denoting greater deviation. Yellow is assigned to residues that could not be matched for alignment. (**c**) Schematic representation of the (i) eIF4a and (ii) RIG-I HEL1 domains. The β-strands are indicated with arrows, α-helices with rods and loop regions with lines. Secondary structures are colored to represent relative proximity to the reader in three-dimensional space, with lighter objects being closer, excluding the helices shown in red, which represent the Pincer helices (Pα1 and Pα2). Positions of pertinent conserved motifs or interaction sites are labeled and marked with arrows.

RIG-I/eIF4a structural alignment was performed using Pymol. Regions to be aligned from the DExD/H-box-containing RecA-like folds of both proteins were selected to include all matching secondary structural elements within the boundaries of these domains. The alignment was performed using the built-in ‘align’ function, and the color was applied using the ‘colorbyrmsd’ script found at http://www.pymolwiki.org/index.php/ColorByRMSD.

## RESULTS

### The pincer domain is required for efficient IFN-β stimulation

To probe the functional importance of the Pincer domain in RIG-I activation, we introduced a series of alanine mutations at interaction sites between the Pincer helices and the helicase core. Three sites at which the Pincer domain engages in electrostatic or hydrophobic contacts with the helicase core were selected for mutation, based on structural data from both human and duck RIG-I constructs (Figure 1a–c). Three amino acids at each site were targeted for mutagenesis, with single, double and triple mutations introduced at sites and residues of particular interest (see Supplementary Table S1). The first site, denoted Pincer 1 mutants, includes residues near the N-terminus of the Pincer that mediate electrostatic interactions with HEL2 (Figure 1c, left panel). Pincer 2 mutants consist of a cluster of residues at the C-terminus of the Pincer that interact near the junction of two α-helices in HEL1 (Figure 1c, center panel). Pincer turn mutants comprise residues spanning the turn between the N- and C-terminal Pincer helices that interact with the linker region between HEL1 and HEL2 and stabilize the inter-helical turn within the Pincer domain (Figure 1c, right panel).

To evaluate the effects of these mutations on RIG-I function, we analyzed the ability of mutant proteins to induce an interferon response in human cells (35,49). Several Pincer 1 and Pincer 2 mutants exhibited varying degrees of impairment in IFN-β induction, whereas Pincer turn mutants behaved much like the WT protein (Figure 1d). Intriguingly, two single mutants at the Pincer 1 site—K750A and N754A—displayed a marked loss in activity when mutated individually, but regained near-WT function in the context of additional mutations at this site (e.g. K750A/E751A and E751A/N754A). We interpret these results to indicate that perturbations in the local electrostatic or hydrogen bonding environment can be partially mitigated by mutations of other nearby residues.

In contrast to mutations at the Pincer 1 site, Pincer 2 mutants exhibited an additive loss in signaling activity as more mutations were accumulated. All single residue substitutions at this site—Q784A, K788A and R791A—resulted in impaired signaling. Diminution in signaling increased for double mutants, and RIG-I was effectively inactivated upon incorporation of alanine residues at all three positions (Figure 1d, center panel). Given the proportional relationship between the number of alanine substitutions at this interface and the relative loss of signaling in cell-based assays, we selected the Pincer 2 mutants for further biochemical analyses.

### The pincer facilitates RNA binding

To evaluate the effects of Pincer 2 site mutations on RNA binding, we employed a fluorescence polarization assay using a fluorescein-labeled, blunt 10mer hairpin that is terminated by a stable UUCG tetraloop, 5′OH10L-F (Figure 2a). Because a considerable proportion of the total binding energy between RIG-I and a triphosphorylated RNA is derived from interactions between the CTD and the triphosphate moiety (50), we elected to use a non-triphosphorylated dsRNA hairpin in this assay in order to sensitize the system and clearly observe changes in energetic contributions from RNA binding by the helicase core.

Recombinant RIG-I proteins were expressed and purified as reported previously (12,35). As a metric for proper folding of the recombinant mutant proteins, we performed stoichiometric binding analyses using RNA concentrations well above *K*_d_. The results show that each mutant is competent for RNA binding and that these mutants bind 5′OH10L-F at the predicted 1:1 ratio (Figure 2b). Importantly, results from this experiment also showed that the WT and mutant proteins were binding within a similar stoichiometric regime, confirming that the percent of folded, active protein in each sample was roughly equivalent. Hence, we assert that any observed defects in binding and catalytic activities we report here are not the result of protein aggregation, degradation or misfolding.

We determined dissociation constants (*K*_d_) by holding the concentration of labeled dsRNA at a constant value that is well below the expected *K*_d_ and titrating Pincer 2 mutants over a 0–1.5 μM range (Figure 2c). For WT RIG-I, we determined a mean *K*_d, 5′OH10L_ of 25 ± 4 nM, which is in good agreement with previously published data on similar ligands (50). Dissociation constants determined for the Pincer 2 mutants ranged from WT values for the single mutants to ∼10-fold weaker for the triple Pincer 2 mutant (*K*_d, 5′OH10L_ = 267 nM), with *K*_d, 5′OH10L_ values for double Pincer 2 mutants falling between these extremes (Figure 2c). A similar trend in the reduction of affinity is seen for the functional binding of Pincer 2 mutants to a triphosphorylated RNA ligand (see *K*_M, RNA, 5′ppp10L_ values, Table 1). We therefore conclude that destabilizing the Pincer region, and more specifically, destabilizing the interaction between the Pincer and HEL1, diminishes the ability of RIG-I to bind duplex RNA. It is important to reiterate, however, that RNA binding by all mutants remains saturable at sufficient RNA concentrations, implying that only the dissociation constant and not the potential for RNA binding has been perturbed in this system.

**Table 1.** Kinetic and binding parameters of Pincer 2 mutants

RIG-I Construct	*k*_cat, ATP, 5′ppp10L_ (s^−1^ RIG-I^−1^)	*K*_M, ATP, 5′ppp10L_ (μM)	*k*_cat, ATP, 5′OH10L_ (s^−1^ RIG-I^−1^)	*K*_M, ATP, 5′OH10L_ (μM)	*k*_cat, RNA, 5′ppp10L_ (s^−1^ RIG-I^−1^)	*K*_M, RNA, 5′ppp10L_ (nM)	*k*_cat, RNA, 5′OH10L_ (s^−1^ RIG-I^−1^)	*K*_M, RNA, 5′OH10L_ (nM)	*K*_d, 5′OH10L-F_ (nM)	Stoichiomety of binding 5′OH10L-F (enzyme/RNA)
Wild Type (WT)	21.04±2.73	303.27±21.77	18.17±0.90	513.57±16.52	19.32±2.11	0.72±0.13	19.09±4.89	12.14±7.48	25.03±3.83	1.28±0.01
Q784A	21.48±1.94	309.83±7.24	21.40±1.11	454.27±33.92	21.28±0.75	1.14±0.10	19.46±0.63	45.53±1.32	23.79±2.90	1.30±0.13
K788A	16.27±1.46	286.50±22.58	16.60±2.06	462.53±40.65	15.88±2.05	0.30±0.17	16.75±3.95	17.96±6.08	21.64 ±8.91	1.27±0.14
R791A	20.74±5.75	309.27±48.42	18.34±3.03	619.87±86.45	14.86±3.52	4.78±1.83	14.93±1.11	63.84±4.32	50.69±16.12	1.42±0.10
Q784A/K788A	6.01±3.18	285.93±71.12	4.99±1.07	390.10±65.05	2.94±0.17	15.36±1.38	3.49±0.29	67.54±1.56	85.85±16.09	1.00±0.06
Q784A/R791A	4.96±4.27	271.17±88.80	3.66±1.71	445.57±59.07	4.81±2.47	12.30±5.90	7.32±0.45	84.67±9.45	91.41 ±21.60	1.59±0.07
K788A/R791A	7.02±2.77	303.50±53.88	5.35±0.38	456.50±70.59	5.94±0.52	9.74±0.55	5.85±0.64	100.23±4.58	166.33±9.07	1.01±0.01
Q784A/K788A/R791A	N/A	N/A	N/A	N/A	N/A	N/A	N/A	N/A	172.37±67.43	1.54±0.10

Kinetic and binding parameters for WT and Pincer 2 mutant RIG-I constructs. Construct designations are listed in the left column, with double and triple substitution mutations separated by a slash. *k*_cat, 5′n10L_: maximal rate of ATP hydrolysis stimulated by a 10mer hairpin bearing a triphosphate (*n* = ppp) or a hydroxyl (*n* = OH) moiety at the 5′ terminus; *K*_M, RNA, 5′n10L_: Michaelis constant for RNA in ATP hydrolysis stimulated by 5′ppp10L or 5′OH10L; *K*_M, ATP, 5′n10L_: Michaelis constant for ATP in ATP hydrolysis stimulated by 5′ppp10L or 5′OH10L; *K*_d, 5′OH10L-F_: equilibrium dissociation constant for binding of fluorescently labeled 5′OH10L. Values represent the combined results of at least three independent experiments and are reported with the standard deviation across these experiments.

### The pincer domain regulates catalytic activity

Having established that the Pincer domain plays a role in optimizing RNA duplex recognition by RIG-I, we next evaluated the consequences of destabilizing the Pincer 2:HEL1 interface on catalytic activity of the enzyme. We measured the steady-state kinetic parameters for RNA-stimulated ATP hydrolysis using a coupled assay system (51). For consistency, ATP hydrolysis by RIG-I was stimulated by a 10mer hairpin identical in sequence to that used in RNA binding experiments. In order to evaluate the relative contribution of the triphosphate moiety to catalysis, we measured ATPase activity in the presence of two different RNA ligands: an RNA bearing a 5′ hydroxyl (5′OH10L) or a 5′ triphosphate (5′ppp10L) (Figure 3a). To examine the effects of Pincer 2 mutants on both the RNA and ATP requirements for RIG-I catalysis, we determined the catalytic rate constants of the enzymes (*k*_cat_) in two regimes: by measuring velocity as a function of varying either RNA or ATP concentration, which allowed the determination of Michaelis constants for each ligand (*K*_M, RNA_ and *K*_M, ATP_, respectively) (Figure 3b and c, Table 1).

A first goal was to determine whether Pincer 2 mutants might influence the catalysis of ATP hydrolysis, causing an apparent reduction in *k*_cat_ values. Introduction of single Pincer 2 mutants did not significantly perturb values for *k*_cat_. However, the introduction of double substitution mutations at the Pincer 2:HEL1 interface greatly reduced *k*_cat_ values, and mutation of all three Pincer 2 residues eliminated detectable ATPase activity altogether (Figure 3b and c). Importantly, RNA ligand saturation was achieved in all of these assays (Figure 2c and Materials and Methods), thus the observed effects are not attributable to any defects in RNA binding. We can therefore conclude that effectively all protein is in a ligand-bound state for each mutant construct, and that the *k*_cat_ reductions observed for Picer 2:HEL1 mutants reflect a diminution in ATP hydrolysis efficiency.

In addition to the effects on maximal rate constants (*k*_cat_ values), Pincer 2 mutants exhibited reductions in *K*_M, RNA, 5′OH10L_ that correlate well with the observed trends in *K*_d_ values (Figures 2c and 3b, Table 1). Specifically, mutants that display weaker dsRNA binding also require higher ligand concentrations to achieve maximal catalytic velocity, in agreement with previous studies showing that *K*_M, RNA_ values obtained under these conditions provide a functional metric for equilibrium dissociation constants (35). As shown previously, RIG-I binds triphosphorylated duplexes more tightly than simple duplexes (*K*_M_, RNA, 5′ppp10L is much lower than *K*_M_, RNA, 5′OH10L, see Table 1). However, despite this enhancement in total affinity, the trends in *K*_M, RNA, 5′ppp10L_ data among Pincer 2 mutants are qualitatively similar to those observed for *K*_M, RNA, 5′OH10L_. Thus, while the disparity between Michaelis constants for triphosphorylated and hydroxyl ligands is consistent with the known preference for a triphosphorylated ligand (12,35), the fact that Pincer 2 mutants disturb the *K*_M, RNA_ for both types of ligands to comparable extents suggests that the gain in binding energy from the CTD:triphosphate interaction is not sufficient to overcome the observed defects of Pincer 2 mutations (Figure 3b, Table 1). Therefore, the observed reductions in apparent RNA affinity are due to a disruption in contacts between the helicase domain and regions of the RNA duplex.

Taken together, these kinetic data indicate that decoupling the pincer from the ATPase core weakens RNA binding and, distinct from this effect, it also disrupts communication between RNA binding modules and the ATPase active site, thereby blocking the transduction of information on viral RNA detection. Given that the ATP binding pocket is located ∼ 30 Å from the nearest Pincer 2 mutant residue, these data suggest that Pincer 2 mutants influence an allosteric network of interactions within the RIG-I protein.

### Pincer: HEL1 decoupling disrupts ATPase activity at the chemical step

Given their dramatic effects on the efficiency of ATP hydrolysis, it was important to determine whether the Pincer 2 mutants influence ATP binding or reaction chemistry. To measure the apparent affinity for ATP, we monitored the velocity of ATP hydrolysis as a function of ATP concentration, obtaining values for *K*_M, ATP_ (Figure 3c, Table 1). Under conditions of saturating RNA 5′OH10L, we obtained *K*_M, ATP, 5′OH10L_ values ranging from 400 to 650 μM, with a mean standard deviation of ∼75 μM. Mean values obtained for each mutant fall within error of the statistical average taken across all datasets, and the number of mutations accumulated has no effect on *K*_M, ATP, 5′OH10L_. Furthermore, data obtained with the 5′ppp10L duplex are consistent with those obtained with 5′OH10L, although the values exhibit a tighter distribution with significantly smaller deviations. For example, the *K*_M, ATP, 5′ppp10L_ values range from around 280 μM to around 340 μM, with a mean error of ∼40 μM.

Based on this data, we observe no statistically significant difference in relative *K*_M, ATP_ values across all Pincer 2 mutants tested for either ligand, suggesting that the ability of the protein to bind ATP remains largely undisturbed across all constructs. Further, because we observe that *k*_cat_ values achieve a plateau within the range of ATP concentrations used in the experiment, we can conclude that providing additional ATP will not yield improvements in catalysis. Taken together, these results show that, after dsRNA binding, all Pincer 2 mutants become equally competent to interact with ATP, but that saturating these proteins with ATP does not rescue WT-like catalytic activity. These findings indicate that although an ATP binding pocket is formed by all Pincer 2 mutants, the orientation of important catalytic residues within that pocket is perturbed in these proteins.

The fact that saturating amounts of RNA and ATP cannot rescue WT-like *k*_cat_ values in Pincer 2 mutants indicates that perturbations in ATPase activity caused by these mutations are independent of ternary complex equilibria and do not directly result from defects in ligand or substrate binding. We can therefore conclude that decoupling of the Pincer 2:HEL1 interaction results in allosteric destabilization of residues involved in the chemical step of ATP hydrolysis, indicating that ATPase core function in RIG-I has become dependent on intramolecular interactions with the Pincer domain.

### Adaptations in RIG-I HEL1 facilitate interactions with the pincer domain

An indirect or allosteric mechanism of catalytic modulation by the Pincer domain is in good agreement with structural data available for RIG-I. As mentioned previously, no residues in the Pincer 2:HEL1 are directly involved in RNA binding, ATP binding or ATP hydrolysis; that is, none of the residues that have been mutated at this interface—nor any of the residues with which they interact—physically contact either dsRNA or the ATP substrate in any of the available RIG-I structures. In an effort to better understand the mechanism by which the Pincer domain might exert control over binding and catalytic activities mediated by other domains of RIG-I, we performed a structural analysis of the RIG-I HEL1 domain by comparing it to homologous domains found in other canonical SF2 proteins.

While the RecA-like domains of most DExD/H-box proteins share a similar core architecture, the primary sequences of these proteins often vary considerably outside of the conserved motifs that are characteristic of all Superfamily 2 (SF2) enzymes (52). Therefore, comparing the tertiary structure of RIG-I HEL1 with structures of homologous domains in prototypical DEAD- and DECH-box proteins was more useful than primary sequence comparison or secondary structure prediction analyses for evaluating unique features of the RIG-I HEL1 fold.

To undertake this analysis, we used the Dali server (http://ekhidna.biocenter.helsinki.fi/dali_server/start) to perform a search against the ATPase core of RIG-I (PDB 4A36) (13,53). Briefly, this server performs a pairwise alignment analysis between a search structure and all structures deposited in the PDB. The server compares the three-dimensional architectures of two structures over a set of residues that can be matched by sequence and secondary structure analyses. We selected best matches identified by the Dali server for secondary structure comparison in order to identify RIG-I subdomains that differ from the conserved DExD/H-box RecA fold.

Based on this analysis, we identified two regions surrounding the sixth β-strand (β6) of RIG-I HEL1 that have little or no overlap among similar folds identified by Dali (Figure 4a). The first region comprises portions of the fifth helix (α5.b) and the adjacent flexible linker that leads into β6, while the second region comprises the linker following β6 and portions of the sixth component helix of HEL1 (α6). To visualize these regions in three-dimensional space, we performed a structural alignment between the RecA folds of RIG-I and the DEAD-box helicase eIF4A. RIG-I HEL1 residues were color-coded based on pairwise residue RMSDs between the RIG-I and eIF4A folds, with deviation between the proteins increasing from blue to green, and yellow residues having no match between the two structures (Figure 4b). As predicted by secondary structural analysis, the most significant tertiary structural deviations occur at α5.b and α6, with α6 being the more pronounced. This helix, along with the adjacent flexible linker that tethers it to β6, includes an ∼12 to 13 amino acid insertion, and it is rotated ∼45° out of register with the otherwise parallel array of secondary structural elements (Figure 4c). Importantly, it is the α5.b and α6 helices that are primarily responsible for interactions with residues at or near the Pincer 2:HEL1 interaction site. Indeed, the α6 helix bisects the Pincer domain like an axel and potentiates a number of interactions that appear to maintain RIG-I architecture.

We therefore propose that the HEL1 domain of RIG-I has diverged and specifically adapted to include interactions with the Pincer domain. Further, the observation that these interactions are now required for optimal catalytic function of the protein may in part be explained by the presence of the conserved SAT motif within the linker between β6 and α6. This motif, found in all SF2 proteins, has been implicated in coordinating RNA binding and ATPase activities of the protein family (54–57). Based on the binding and catalytic deficiencies that result from Pincer 2:HEL1 perturbations, we conclude that interactions between the Pincer and modified secondary structural elements of HEL1 provide stability within the active site by orienting important residues required for catalysis, and by propagating the allosteric effects of dsRNA binding.

## DISCUSSION

In an effort to investigate the role of the Pincer domain in RIG-I signaling, we produced a series of alanine substitution mutants that were predicted to disrupt interaction sites between the Pincer and other domains of the enzyme. We found that perturbations at the N- and C-termini of the Pincer domain impaired signaling activity, and that this behavior was exacerbated by accumulated mutations at the Pincer 2:HEL1 interface. Using recombinant RIG-I proteins belonging to the Pincer 2:HEL1 subset, we have shown that disrupting the interaction between these domains results in decreased affinity for dsRNA and a decreased maximal rate constant for ATP hydrolysis. We have also demonstrated that the HEL1 domain of RIG-I is structurally distinct from homologous domains of related DExD/H-box proteins, and that the novel helical arrangement of this domain provides specialized interfaces for mediating interactions between HEL1 and the Pincer. Based on these collective data, we propose that the Pincer has become an essential architectural component of the RLR core, and that it serves to link RNA binding to productive catalysis.

The observation that Pincer 2:HEL1 mutants exhibit reduced levels of ATPase activity under saturating ligand and substrate concentrations implies a physical perturbation at the active site that cannot be overcome by either RNA or ATP binding. Intriguingly, despite these catalytic defects, mutants do not display reduced *K*_M, ATP_ values. This suggests that interaction between the ATPase core and the nucleotide substrate remains undisturbed among the Pincer 2:HEL1 mutants, but that subsequent chemical events required for hydrolysis no longer proceed efficiently. This behavior—similar to that observed in other DEAD-box proteins bearing mutations in the conserved SAT motif—is indicative of a break in the link between RNA binding and ATP hydrolysis (56,57).

Pincer 2:HEL1 mutants also display reduced affinities for RNA, yielding increased *K*_M, RNA_ values for 5′ hydroxylated and 5′ triphosphorylated hairpins compared to WT RIG-I. Because the trends in *K*_M, RNA_ are consistent for both RNAs, we favor an interpretation in which dsRNA binding is perturbed through a diminished contribution by the ATPase fold and specifically the HEL1 domain.

Taken together, these results suggest that productive organization of multiple interfaces within the DExD/H-box core relies upon architectural contributions from the Pincer domain. The alpha helices of the Pincer appear to be appended to a DExD/H-box core that is no longer capable of functioning on its own, outside of the unique architectural context that has evolved among RLR proteins. We propose that the modified secondary structure and architecture of HEL1 may enhance the sensitivity of RIG-I as a specific RNA sensor by imposing a dependence on other domains of the molecule. Specifically, RNA stimulated ATPase activity is dependent on HEL1; HEL1 cannot function without the Pincer; and the Pincer links the ATPase core to the triphosphate recognition domain (CTD). This arrangement applies selective pressure on RIG-I and other RLRs for the maintenance of domains that are appended to what might otherwise be a functionally independent DExD/H-box protein.

In agreement with the hypothesis that RLR Pincer domains attenuate the functions of these surveillance proteins, evolutionary analyses of RLRs in humans and other mammals have shown that sites in the modified α6 helix of HEL1 and in both Pincer helices are regions under positive, or adaptive, selection (58,59). Whether these sites have been selected for their allosteric contributions to intramolecular communication or whether the external face of the Pincer domain represents an interaction site for additional endogenous or viral factors remains an intriguing question. Whatever the mechanism, it is clear that the RIG-I Pincer domain has evolved to play an integral role in converting a basic ATPase core into a sensitive biosensor for detection and response to viral RNA.

## SUPPLEMENTARY DATA

Supplementary Data are available at NAR Online.

SUPPLEMENTARY DATA
