# Supplementary material for: The RIG-I ATPase core has evolved a functional requirement for allosteric stabilization by the Pincer domain
Source: Nucleic Acids Res. 2014 Sep 12;42(18):11601–11. doi: 10.1093/nar/gku817 (PMC4191399; doi:10.1093/nar/gku817)
Supplement: SUPPLEMENTARY DATA [file supp_42_18_11601__index.html]

The RIG-I ATPase core has evolved a functional requirement for allosteric stabilization by the Pincer domain — SUPPLEMENTARY DATA 

# The RIG-I ATPase core has evolved a functional requirement for allosteric stabilization by the Pincer domain

## SUPPLEMENTARY DATA

**Files in this Data Supplement:**

- SUPPLEMENTARY DATA
