# Supplementary material for: The RIG-I ATPase core has evolved a functional requirement for allosteric stabilization by the Pincer domain
Source: Nucleic Acids Res. 2014 Sep 12;42(18):11601–11. doi: 10.1093/nar/gku817 (PMC4191399; doi:10.1093/nar/gku817)
Supplement: SUPPLEMENTARY DATA [file supp_gku817_nar-01787-m-2014-File006.pdf]

RIG-I Pincer Domain Alanine Substitution Mutation Groups and Sites

	Single Mutants	Double Mutants	Triple Mutants
**Pincer 1**	K750A<br>E751A<br>N754A	K750A/E751A<br>E751A/N754A	N750A/E751A/N754A
**Pincer Turn**	Q769A<br>W771A<br>E773A	Q769A/E773A	
**Pincer 2**	Q784A<br>K788A<br>R791A	Q784A/K788A<br>Q784A/R791A<br>K788A/R791A	Q784A/K788A/R791A
